# Fabrication of Silver Nanoparticles Using *Cordyline fruticosa* L. Leave Extract Endowing Silk Fibroin Modified Viscose Fabric with Durable Antibacterial Property

**DOI:** 10.3390/polym14122409

**Published:** 2022-06-14

**Authors:** Ngoc-Thang Nguyen, Thi-Lan-Huong Vo

**Affiliations:** 1 Department of Textile Material and Chemical Processing, School of Textile-Leather and Fashion, Hanoi University of Science and Technology, 1 Dai Co Viet, Hanoi 11615, Vietnam; 2 Department of Fibre and Textile Technology, Hanoi Industrial Textile Garment University, Hanoi 12411, Vietnam; huongvtl@hict.edu.vn

**Keywords:** *Cordyline fruticosa* L., silver nanoparticles, antibacterial activity, silk fibroin, viscose fabric

## Abstract

The current work presented a green synthetic route for the fabrication of silver nanoparticles obtained from aqueous solutions of silver nitrate using *Cordyline fruticosa* L. leaf extract (Col) as a reducing and capping agent for the first time. The bio-synthesized silver nanoparticles (AgCol) were investigated using UV–visible spectroscopy (UV–vis), transmission electron microscopy (TEM), X-ray diffraction (XRD), Fourier transform infrared spectroscopy (FTIR), and thermal gravimetric analysis (TGA). The obtained data demonstrated that AgCol in spherical shape with an average size of 28.5 nm were highly crystalline and well capped by phytocompounds from the Col extract. Moreover, the bio-synthesized AgCol also exhibited the effective antibacterial activities against six pathogenic bacteria, including *Escherichia coli* (*E. coli*), *Pseudomonas aeruginosa* (*P. aeruginosa*), *Salmonella enterica* (*S. enterica*), *Staphylococcus aureus* (*S. aureus*), *Bacillus cereus* (*B. cereus*) *and Enterococcus faecalis* (*E. faecalis*). The AgCol were applied as an antibacterial finishing agent for viscose fabric using a pad-dry curing technique. The AgCol-treated viscose fabrics exhibited a good synergistic antimicrobial activity against *E. coli* and *S. aureus* bacteria. Furthermore, the silk fibroin regenerated from *Bombyx mori* cocoon waste was utilized as an ecofriendly binder for the immobilization of AgCol on the viscose fabric. Thus, the antimicrobial efficacy of the AgCol and fibroin modified viscose fabric still reached 99.99% against the tested bacteria, even after 30 washing cycles. The colorimetric property, morphology, elemental composition, and distribution of AgCol on the treated fabrics were investigated using several analysis tools, including colorimetry, scanning electron microscopy (SEM), energy-dispersive X-ray spectroscopy (EDX), atomic absorption spectroscopy (AAS), Kjeldahl, and FTIR. Because of the excellent antimicrobial efficiency and laundering durability, as well as the green synthesis method, the AgCol and fibroin modified viscose fabric could be utilized as an antibacterial material in sportswear and medical textile applications.

## 1. Introduction

Cellulosic textiles, such as cotton and viscose, play key roles in the daily life of humans due to their excellent properties, including comfortability, water absorptivity, air permeability, and flexibility [1,2,3]. However, this natural material also provides a favorable medium for the adsorption, survival, and growth of hazardous microorganisms owing to its moisture, oxygen, and nutrient retaining ability [2,4,5,6]. Infestation by microbes can cause detrimental effects for the wearer and the textile manufacturing process, such as the generation of unpleasant odor, health concerns, loss of fabric strength properties, and discoloration [2,6]. To overcome those problems, antibacterial finishing of cellulosic textile products is often inevitable. In addition, viscose fabric absorbs more sweat than cotton when worn next to the skin, providing a convenient environment for bacterial growth. Therefore, it is necessary to improve the antibacterial properties of viscose fabric in order to make this cellulosic material more attractive and to broaden its application fields.

Historically, numerous antimicrobial agents, such as quaternary ammonium compounds [7,8], triclosan [7,9], halogenated phenols [7], inorganic nanoparticles [9,10,11], chitosan and its derivatives [1,5,6,12], and their finishing techniques, have been applied for cellulosic fabrics with respect to the effective control of microbial growth [7,13,14]. However, some antimicrobial agents that are in consideration are not eco-friendly, are toxic to humans, and there is a risk that bacteria may grow resistant to the antibiotics [6,14,15]. Thus, the proper selection of antibacterial agent and application process to impart durable antibacterial efficiency to the cellulosic materials not only enables the production of highly active cellulosic products against undesirable bacterial, but also ensures minimal negative impacts on the fabric and environment. In view of these applicable and environmental concerns, the cellulosic fabrics treated with green silver nanoparticles (AgNPs) which were synthesized using bio-reductants from natural resources, i.e., plants, algae, and microorganisms, have greatly focused scientists’ attention due to their wide-ranging antibacterial activity and durability to microorganisms [16,17,18,19,20]. Moreover, AgNPs are associated with low toxicity with regard mammalian cells, and their cytotoxicity can be reduced when they are incorporated into a polymer matrix [16,17,18,21].

One of the utmost important aspects for antibacterial textiles in terms of maintaining hygiene is laundering durability [1,3,17]. Thus, strategies for the immobilization of AgNPs on cellulosic textiles have been a fashion research trend in the modification of textiles. Numerous techniques for this purpose have been developed, including ex situ and in situ fabrication of AgNPs onto the fabric surfaces with the assistance of crosslinking agents or polymer binders [5,22,23,24,25,26,27,28,29,30,31,32]. Among polymer binders, natural polymers, such as chitosan and its derivatives, and starch, gelatin, alginate, pectin, guar gum, and rubber are reported to be very effective and ecofriendly for adhering AgNPs onto cellulosic fabric surfaces [5,27,28,29,31,32]. Interestingly, these polymers have great capacity to suffer bounding to the cellulose molecules of cellulosic fabrics even in very small added amounts. Therefore, they enhance the non-leaching of AgNPs during washing cycles.

Beyond the above knowledge in mind, the present work proposed a new strategy to impart antibacterial functionality to viscose fabric using bio-synthesized AgNPs and regenerated silk fibroin as an eco-friendly polymer binder. Firstly, a green synthesis of AgNPs from a water-based solution of silver nitrate utilizing *Cordyline fruticosa* L. leaf extract as a reducing and capping agent was reported for the first time. *Cordyline fruticosa* L. comprises of more than 480 species distributed mostly in tropical and subtropical regions of the world [33,34,35]. It can be found in gardens as an ornamental plant, as its leaves are very conspicuous and attractive. It has been used as a traditional medicinal plant for centuries in Vietnam. According to the literature, the major constituents present in the *Cordyline fruticosa* L. leaf extract are reported to contain anthocyanin, saponin, flavonoids, flavonols, chlorophyll, glycoside, etc., which possess strong reducing power [33,34,35,36,37,38,39]. The reduction reaction of silver ions was controlled using UV–vis spectroscopy to optimize the synthesis parameters of the AgNPs. The obtained AgNPs were examined using HR-TEM, XRD, FTIR, and TGA analyses. The antibacterial action of the bio-synthesized AgNPs was evaluated against six pathogenic bacteria, including *Escherichia coli* (*E. coli*), *Pseudomonas aeruginosa* (*P. aeruginosa*), *Salmonella enterica* (*S. enterica*), *Staphylococcus aureus* (*S. aureus*), *Bacillus cereus* (*B. cereus*), and *Enterococcus faecalis* (*E. faecalis*). Afterwards, the bio-synthesized AgNPs were applied as an antibacterial finishing agent for viscose fabrics using a pad-dry curing technique. In order to enhance the laundering durability, silk fibroin regenerated from *Bombyx mori* cocoon waste was used as an ecofriendly binder for the pre-modification of viscose fabric and subsequent loading of the AgNPs using the pad-dry curing technique, under different treatment conditions. Finally, the antibacterial activity of the functionalized viscose fabrics against both the gram-negative bacteria *E. coli* and gram-positive *S. aureus* bacteria were assessed after washing cycles. The mechanism of the modification of viscose fabric was suggested. To the best of our knowledge, the regenerated silk fibroin combined with AgNPs in the antimicrobial finishing of viscose fabric has not yet been reported.

## 2. Materials and Methods

### 2.1. Materials

*Cordyline fruticosa* L. leaves (Col) were collected from a locality of the Hoai Duc district, Hanoi, Vietnam, in October 2019. First of all, the Col leaves were washed with distilled water, before being dried at 60 °C for 48 h. The dried leaves were thinly chopped into small pieces (5 × 5 mm) and kept in a sealable plastic bag for further study. *Bombyx mori* cocoon waste was collected from the village of Vong Nguyet, Bac Ninh province, Vietnam. Plain woven viscose fabric (staple viscose, Ne30/1, 68 ends/cm, 68 picks/cm) was scoured and supplied by the Nam Dinh Textile Garment Co., Ltd., Nam Dinh, Vietnam. Silver nitrate (AgNO_3_, 99.99%), sodium carbonate, ethanol, acetic acid, lithium bromide, and aluminum sulfate octadecahydrate were purchased from the Aladdin Shanghai Biochemical Technology Co. Ltd., Shanghai, China. The solvent used in all experiments was double distilled water from an EYELA Still Ace SA-2100E. Six types of bacteria, including *Escherichia coli* (ATCC 8739), *Pseudomonas aeruginosa* (ATCC 27853), *Salmonella enterica* (ATCC 2162), *Staphylococcus aureus* (ATCC 25923), *Bacillus cereus* (ATCC 11778), and *Enterococcus faecalis* (ATCC 29212) were obtained from the Center for Research and Development in Biotechnology, HUST, Hanoi, Vietnam.

### 2.2. Preparation of Cordyline fruticosa L. Leaf Extract

**Figure 1:**
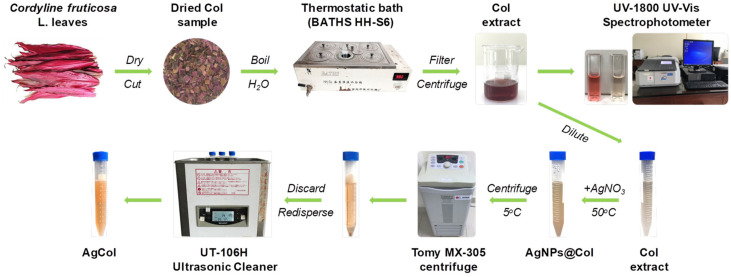
Schematic representation of the *Cordyline fruticosa* L. leaf extraction by the maceration method and the phytochemical-mediated synthesis of AgCol from the extract.

The dried Col leaf sample weighing up to 10 g was boiled in a 500 mL triangular flask containing 200 mL double distilled water for 10 min. The extract solution was filtered using a Whatman No.1 filter paper, then centrifuged at 10,000 rpm for 20 min. The obtained Col supernatant was analyzed using the pH differential method to determine the total anthocyanin content (TAC) of the Col extract [33,40]. The anthocyanin was a main component in the aqueous extraction of Col leaves [33,34], hence its concentration was adjusted to control the reducing power of the Col extract in the synthesis reaction of AgCol. The Col extract was then diluted to an anthocyanin concentration of 10 mg/L by adding double distilled water, before being using for the synthesis of AgCol, where it was kept stored at 4 °C for further use. A schematic illustration on preparation of Col leaf extract by the maceration method was represented in Figure 1.

### 2.3. Synthesis of Silver Nanoparticles

The silver nanoparticles were prepared by adding 1 mL of aqueous silver nitrate solutions at varying concentrations (6, 10, 14, and 18 mM) to 10 mL of the diluted Col extract (10 mg/L anthocyanin) in Falcon tubes. The reaction mixtures were kept in the dark at 50 °C with different incubation times (2, 4, 6 and 8 h). After the passage of time, the synthesized nanoparticles were centrifuged at 15,000 rpm for 30 min at 5 °C. The solution was discarded, and the obtained pellet was re-dispersed in the double distilled water using a sonication bath (UT-106H Ultrasonic Cleaner, Japan). This step was repeated two times to remove residual reagents, and the purified nanoparticle solution (AgCol) was stored at 4 °C prior to analysis. A schematic illustration of the phytochemical-mediated synthesis of AgCol from Col extract was illustrated in Figure 1.

### 2.4. Dissolution of Silk Fibroin

**Figure 2:**
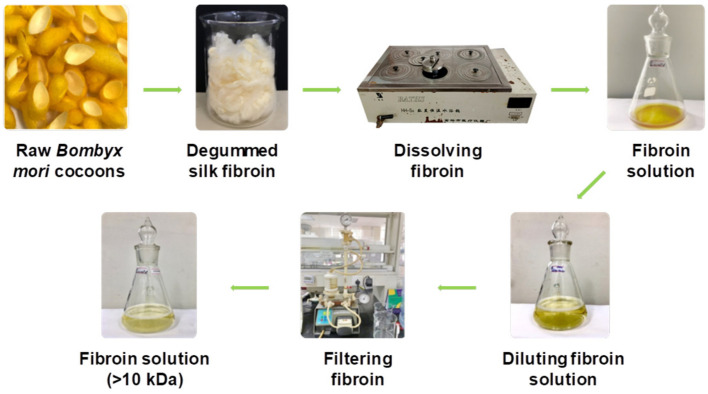
Schematic illustration of degumming, dissolving, and filtering silk fibroin.

The dissolution of silk fibroin procedure was reported in our previous work [41]. In a typical experiment, *Bombyx mori* cocoons were degummed by boiling in an aqueous solution containing 5 g/L Na_2_CO_3_ at a liquor ratio of 1:20 (mass in gram per volume in mL) for 30 min. The silk fibroin was rinsed several times using warm and cold distilled water, and then neutralized with acetic acid (2 mL/L). The sample was washed again in plenty of distilled water and dried at 60 °C until at a constant weight. After that, 2.8 g degummed silk fibroin was dissolved in a glass flask containing 10 mL solution of lithium bromide/ethanol/water (LiEtW) with a mass ratio of 45:44:11 at 80 °C for 60 min. The obtained fibroin solution was diluted 15 times with double distilled water to reduce the viscosity. Excess LiBr and ethanol were then removed from the fibroin solution through microfiltration and ultrafiltration systems with hollow-fiber cartridges in the QuixStand Benchtop system (GE Healthcare, Chalfont Saint Giles, UK). In the first stage of the filtering process, 0.2 µm hollow-fiber cartridge was used to remove impurities and high molecular weight fibroin segments. In the next stage, the fibroin solution was subsequently filtered through a 10,000 NMWC (nominal molecular weight cutoff) hollow fiber ultrafiltration cartridge to get the fibroin segments with molecular weight over 10 kDa retaining inside the filter tube. The solution passed through the ultrafiltration system contained low molecular weight fibroin segments, excess LiBr, ethanol, and water. The fibroin content in the obtained solution was measured using an infrared moisture analyzer (MA35, Sartorius AG, Göttingen, Germany). The scheme of degumming, dissolving, and filtering silk fibroin was illustrated in Figure 2.

### 2.5. Modification of Viscose Fabrics with Silk Fibroin and Silver Nanoparticles

Silk fibroin treated viscose fabric (VisFib) was prepared following our previously reported method [41]. Briefly, the viscose fabric samples with size of 35 × 35 cm were padded twice using an Atlas D394A laboratory padder to achieve a wet pickup of 80% with the 2.5% fibroin solutions (Fib > 10 kDa). The fabrics were then dried at 110 ± 3 °C for 2 min using SDL mini-drier 398 laboratory thermo-fixation. Subsequently, the dried samples were soaked in a 10 g/L aluminum sulfate solution, padded at 80% wet pickup, and cured at 110 ± 3 °C for 5 min to regenerate and fix silk fibroin onto viscose fabrics. The samples were rinsed to remove unfixed and excess reactants and dried again.

**Figure 3:**
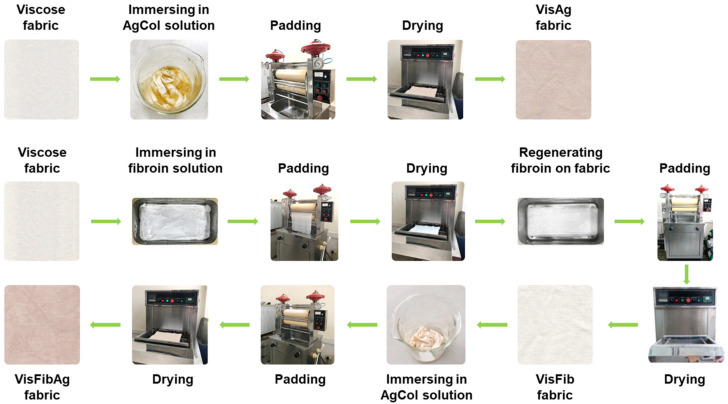
Scheme of modification of viscose fabrics with silk fibroin and AgCol.

In the next step, the untreated and treated viscose fabrics were individually immersed in serial AgCol solutions with different concentrations of 80, 40, and 20 μg/mL for 15 min at a liquor-to-fabric ratio of 5:1 (*w*/*w*) and squeezed using the Atlas D394A laboratory padder to allow various wet pickups of 70%, 80%, and 90%. After that, the padded fabrics were dried at 110 ± 3 °C for 2 min using SDL mini-drier 398 laboratory thermo-fixation. The dipping–padding–drying processes of the fabric samples were repeated two times. The name of AgCol embedded fabric samples was denoted in Table 1. The process of viscose fabrics treated with silk fibroin and AgCol was depicted in Figure 3.

### 2.6. Analytical Methods

#### 2.6.1. The Total Anthocyanin Content of the Col Extract

The total anthocyanin content (TAC) of the Col extract was measured via the pH differential method using two buffer systems of pH 1.0 (KCl + HCl) and pH 4.5 (KHC_8_H_4_O_4_ + HCl). The absorbance of the samples was recorded between 400 and 700 nm in a UV–vis spectrophotometer (UV-1800, Shimadzu, Japan) at a resolution of 1 nm, in 10 mm optical path length quartz cuvettes. The TAC (mg cyanidin-3-glucoside (C3G)/L) of samples was calculated via the following Equation (1) [33,40]:

TAC (mg/L) = A × M × DF × 1000/(ε × d)(1)

where 

A (absorbance) = (A_λmax_ − A_700nm_)_pH=1.0_ − (A_λmax_ − A_700nm_)_pH=4.5_;

M (molecular weight) = 449.2 g/mol for C3G;

DF (dilution factor);

1000 = conversion from g to mg;

ε (molar absorptivity coefficient in L/mol/cm for C3G) = 26,900;

d (path length) = 1 cm.

#### 2.6.2. Characterization of the AgCol

The optical absorption spectra of the AgCol were observed using a UV–vis spectrophotometer (UV-1800, Shimadzu, Japan) between 300 and 700 nm.

Transmission electron microscopy (TEM, JEOL JEM-1400, Tokyo, Japan) was employed to identify the morphology and size of the AgCol.

The XRD patterns of nanoparticles were recorded on a powder X-ray diffractometer (XRD, Oxford Instruments, Oxford, UK) using CuKα radiation (λ = 1.541 Å), operating at 40 kV and 40 mA, in the range of 10° ≤ 2θ ≤ 80°. The particle size of AgCol was calculated using the Debye–Scherrer Equation (2).

(d = 0.91λ/βcosθ)(2)

Fourier transform infrared spectra of the Col extract and the AgCol were obtained with a FTIR spectrometer (Nicolet 6700, Thermo Scientific, Waltham, MA, USA) in the range of 4000–500 cm^−1^.

Thermogravimetric analysis of the AgCol was carried out on a thermo gravimetric analyzer (TGA-209F, Netzsch, Selb, Germany). The sample was heated to temperature ranging from 40 °C to 800 °C under N_2_ atmosphere, at 10 °C/min heating rate.

#### 2.6.3. Characterization of the Modified Viscose Fabrics

A scanning electron microscope (SEM, SM-6510LV JEOL, Japan) coupled with an energy dispersive X-ray spectroscope (EDX, Oxford EDS Microanalysis System, Oxford, UK) was used to determine the morphologies and elemental compositions of the untreated and treated viscose fabrics with Fib and AgCol after platinum sputtering.

The FTIR measurements of the untreated and treated viscose fabrics with Fib and AgCol were studied via a FTIR spectrometer (Nicolet 6700, Thermo Scientific, USA).

The Kjeldahl method (Gerhardt Vapodest Kjeldahl Analysis System, Germany) was employed to detect the content of Fib in the modified viscose fabrics according to the AOAC Official Method 2001.11 with the conversion factor of 6.25 [42].

Silver contents in the modified viscose fabrics were measured using a Atomic Absorption Spectrometer (AAS, PinAAcle 900T, PerkinElmer, Waltham, MA, USA).

Color changes of the modified viscose fabrics, in terms of L*, a*, and b* values, and color differences (ΔE*) were determined using a reflectance spectrophotometer (Ci4200, X-rite, USA) with D65 illumination, and a 10° observer. In the CIELab color space, L* represents lightness, while a* and b* represent chromaticity parameters. The average color parameter values were evaluated at three positions for each sample. The overall color change was calculated based on the following Equation (3):

(3) $$\Delta E=\sqrt{\Delta L^{*2}+\Delta a^{*2}+\Delta b^{*2}}$$

where ΔL*, Δa*, and Δb* represent the changes in L*, a* and b* between the initial and three values, respectively.

### 2.7. Antibacterial Activities

#### 2.7.1. Bio-Synthesized Silver Nanoparticles (AgCol)

The antibacterial activities of the AgCol against three gram-negative bacteria (*E. coli*, *P. aeruginosa* and *S. enterica*) and three gram-positive bacteria (*S. aureus*, *B. cereus* and *E. faecalis*) were studied by using well diffusion assay with the Clinical Laboratory Standard Institute guidelines [43]. In a typical experiment, each bacterial strain was sub-cultured in nutrient broth at 37 ℃ until it reached a count of approximately 10^8^ colony-forming units (CFU) per mL in sterile screw-cap test tubes. Afterwards, each bacterial suspension was spread uniformly on the individual agar petri plates by using sterile cotton swabs. Seven wells with the diameter of 6 mm were aseptically punched using a sterile cork borer, and then 30 μL of Col extract (10 mg C3G/L), AgCol solutions at different concentrations (120, 60, 30 and 15 μg/mL), a standard antibiotic (Chloramphenicol, 200 μg/mL) as positive control, and double distilled water as a negative control were poured into their respective wells. These plates were incubated at 37 °C for 24 h, and the obtained zones of inhibition (ZOI) were measured using the following Equation (4):

W = (T − D)/2(4)

where

W: width of clear zone of inhibition, mm;

T: total diameter of the test specimen and clear zone, mm;

D: diameter of the test specimen, mm.

#### 2.7.2. The Modified Viscose Fabrics with AgCol and Silk Fibroin

The antibacterial activities of the untreated and treated fabrics with AgCol and silk fibroin against both *E. coli* and *S. aureus* were evaluated qualitatively and quantitatively according to the established protocols to test the antibacterial activity of textiles. The following methods were used: AATCC 147-2004 and ASTM E2149-10 test methods.

For the parallel streak method AATCC 147-2004, the rectangular fabric samples (15 × 55 mm) were prepared and pasteurized by the autoclave at 121 °C for 20 min. About 1 mL of each strain culture grown overnight brain heart infusion broth (BHI), at approximately 10^7^ CFU/mL, was mixed with 9 mL of sterile distilled water. Then, one loopful of diluted inoculum was transferred to the surface of the sterile agar plate by making five streaks, approximately 60 mm in length, spaced 10 mm apart and covering the central area of a standard petri dish. The prepared fabric samples were gently pressed by contact across the five inoculum streaks on the agar surface and incubated at 37 °C for 24 h. The width of the clear zone of inhibition was used to determine the antimicrobial activity.

For the dynamic shake flask method ASTM E2149-10, about 1 g of each fabric sample was cut into small pieces and transferred into individual sterile glass flasks containing 50 mL of bacterial suspension with a concentration of 10^5^ CFU/mL. All flasks were loosely capped, placed in an incubator, and shaken at 37 °C and 120 rpm using an incubator shaker for 1 min and 1 h. Afterwards, a series of dilutions of the bacterial sample solutions using a buffer solution and 100 μL was spread over the agar plate. Bacteria recovery at after 1 min (zero contact time) and 1 h were determined. The inoculated plates were then incubated at 37 °C for 24 h and the surviving bacteria were counted. The percent reduction of bacteria was calculated using the following Equation (5):

R = (B − A) × 100%/B(5)

where

R: the bacterial reduction percentage, %;

A and B: the number of bacteria recovered for the flasks containing test samples and without samples (blank), respectively.

Each experiment was performed in triplicates and the final values were presented as the mean ± standard deviation (SD).

#### 2.7.3. Durability of the Antimicrobial Treatment against Washing

To evaluate the durability of the antimicrobial treatment, the washing fastness test on the modified viscose fabrics was carried out in accordance with the AATCC 61-2013 (2A) standard, which simulates five home laundering cycles. The stability of the treated viscose fabric with AgCol and Fib was examined through antibacterial tests against *E. coli* and *S. aureus* after 5, 10, 20, and 30 washes in the presence of a non-ionic detergent.

## 3. Results and Discussion

### 3.1. Synthesis and Optimization of AgCol from Col Extract

**Figure 4:**
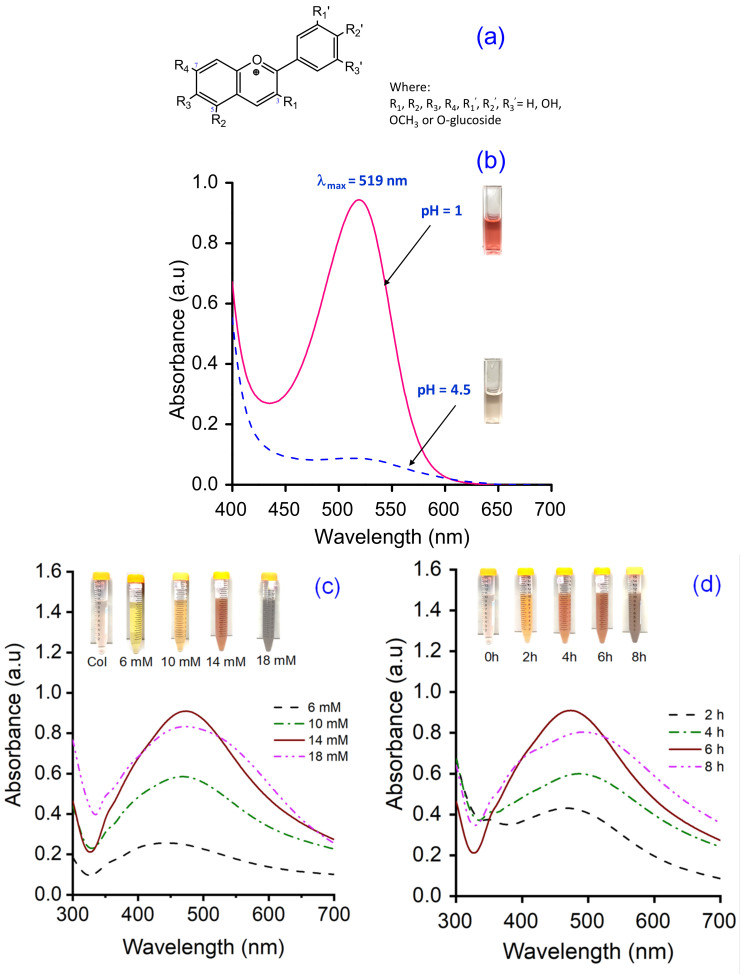
(**a**) The molecular structure of anthocyanin, (**b**) UV–vis spectrum of Col extract, UV–vis spectra of AgCol synthesized at (**c**) different AgNO_3_ concentrations, and (**d**) reaction times. Real images of the samples are shown as insets in (**b**–**d**).

Previous reports confirmed that biological compounds existing in plant extracts, such as alkaloids, flavonoids, polysaccharides, anthocyanins, glycosides, proteins, could played a dual role of both reducing and stabilizing agents for the synthesis of AgNPs [18,20,44,45,46]. In addition, the size, shape, and yield of bio-synthesized AgNPs depend not only on the content of bio-compounds but also on the type of compounds present in the plant extract [45,46]. The *Cordyline fruticosa* L. leaf was reported to contain anthocyanin, saponin, flavonoids, flavonols, chlorophyll, glycoside, etc., in which anthocyanin was one of the major compounds of its aqueous extract [33,34,35,36,37,38,39]. The primary molecule structure of anthocyanin is cyanidin-3-glucoside, which was depicted in Figure 4a. Based on the data obtained from the UV–vis spectra of Col extract at pH = 1.0 and pH = 4.5 (Figure 4b), the TAC of the Col extract was calculated to be 109.72 ± 0.63 mg C3G/L. Anthocyanin belongs to polyphenol compounds, which have inherent reduction property. In this research, the anthocyanin content of the Col extract was adjusted to control the reducing power of the Col extract in the green synthesis reaction. For the AgNPs synthesis, the Col extract was diluted to a fixed concentration of 10 mg C3G/L, mixed with the silver nitrate solution at various concentrations, and kept at 50 °C for different periods of reaction time.

The synthesis of AgNPs was investigated in various conditions, such as AgNO_3_ concentration and reaction time, which were identified as factors affecting the properties and yields of AgNPs. As seen from Figure 4c,d, the addition of AgNO_3_ solution into the Col extract exhibited a color change from faint pink to yellowish-brown, indicating the excitation of surface plasmon resonance (SPR) during the reduction of silver ion to metallic silver [47]. The UV–vis absorption spectra of AgCol solutions showed the SPR peaks at around 470 nm, which confirmed the formation of the AgNPs. As depicted in Figure 4b,c, the Col extract did not show the SPR peak in the visible range, while the apparent SPR peak at 436 nm for 6 mM AgNO_3_ concentration was exhibited. A relative red-shift of maxima of the SPR peak upon increasing the concentration of AgNO_3_ can be noticed from 436 to 478 nm. This red-shift of the peak could be due to the enlargement of AgNPs [15]. In addition, the peak intensity reached its maximum at a wavelength of 470 nm for the 14 mM AgNO_3_ concentration, and then it decreased with higher concentrations. The decreasing and flattening of the resonance peak at AgNO_3_ concentration of 18 mM could be due to the lower yield and larger size of AgCol [15,19,47,48]. Based on the obtained results, 14 mM AgNO_3_ was fixed for further optimization of other parameters. To evaluate the effect of reaction time on the AgCol synthesis, the UV–vis spectra were recorded at 2, 4, 6, and 8 h. As shown in Figure 4d, the SPR peak intensities of AgCol were noted to increase with a longer reaction time. However, a single, sharp, and highest peak absorbance was observed at 470 nm after a 6 h reaction, which provided evidence for spherical-shaped AgCol with a narrow particle size range [48,49,50]. In congruence with experimental observation, the optimum conditions for the bio-synthesis of AgCol were 14 mM AgNO_3_ solution in a 1:10 ratio of the diluted Col of 10 mg C3G/L, and a reaction time of 6 h.

### 3.2. Morphological and Structural Characterization of AgCol

**Figure 5:**
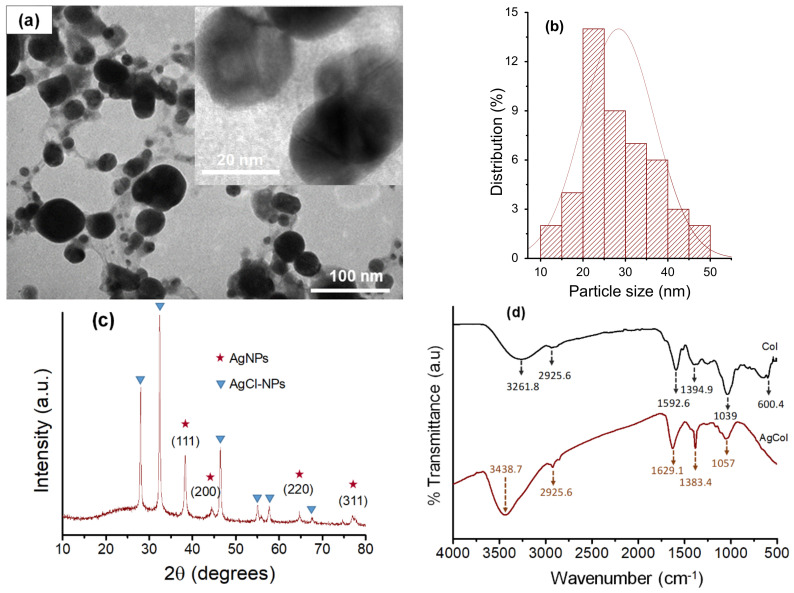

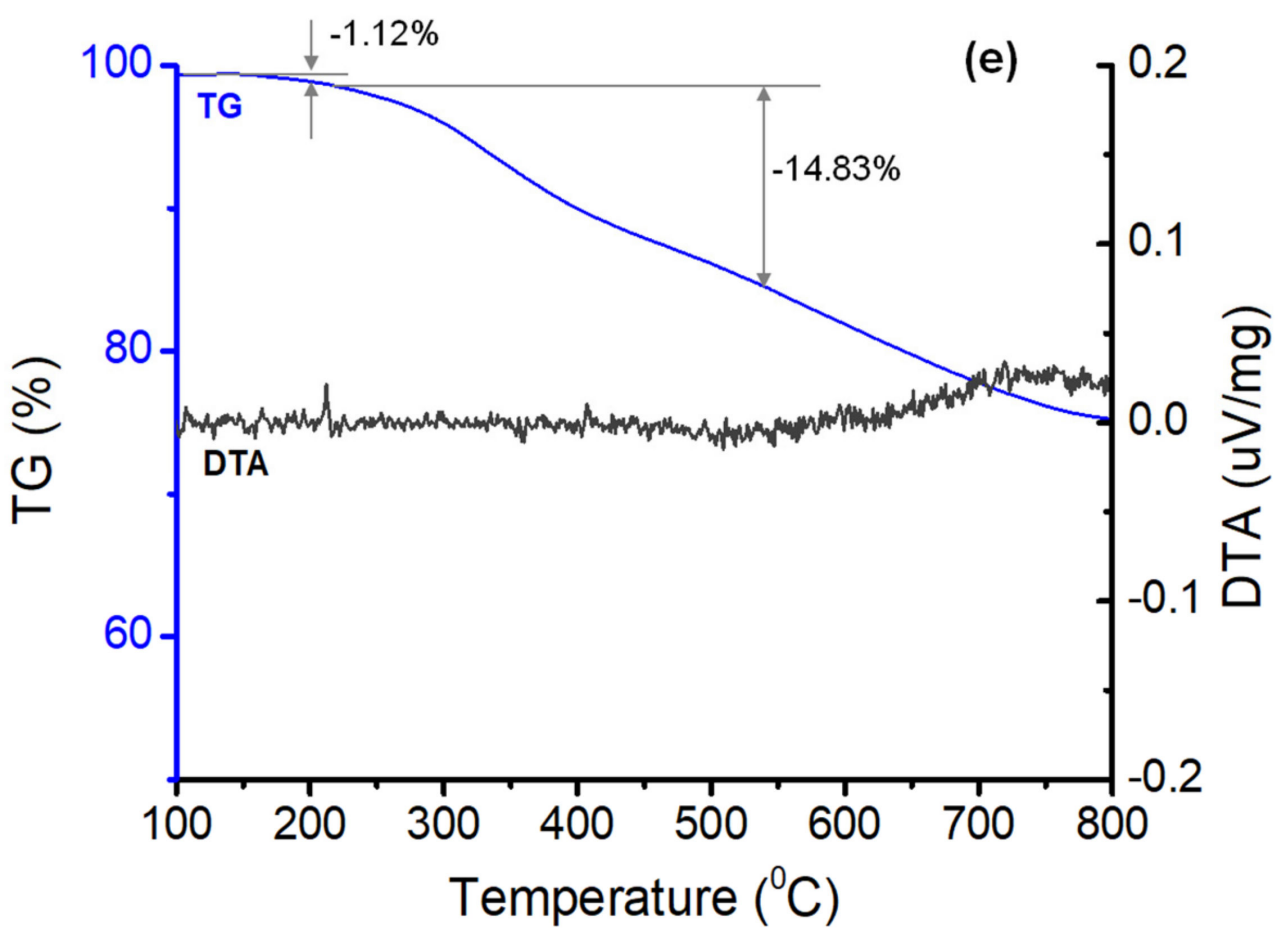
(**a**) TEM micrographs of AgCol with different magnification ×40 k and ×500 k (inset), (**b**) histograms of the AgCol size distribution; (**c**) XRD pattern of AgCol, (**d**) FTIR spectra of Col and AgCol, and (**e**) TGA of AgCol.

The TEM measurement was carried out to study the morphology and size of the AgCol. Figure 5a,b revealed spherical-shaped nanoparticles with a size of 10–50 nm in diameter with an average size of 28.5 nm. The spherical-shaped nanoparticles produced in the current study are in line with the expected shapes for AgCol. This result is consistent with the UV–vis observation and previous reports [44,45,48].

The crystalline structure of the AgCol was studied by recording the XRD pattern. As shown in Figure 5c, the XRD pattern of the AgCol revealed both the diffraction peaks of AgNPs and silver chloride nanoparticles. The diffraction peaks were observed at 2θ = 38.18° (111), 43.72° (200), 64.45° (220), and 77.72° (311), confirming that the AgNPs were crystalline in nature and had the face center cubic (FCC) structure of metallic silver [45,51]. Other diffraction peaks at 2θ = 27.90° (111), 32.30° (200), and 46.00° (220) were corresponded to silver chloride nanoparticles (JCPDS No.: 85-1355) with FCC geometry [52,53,54]. These silver-based nanoparticles are often found in the final product of plant-mediated green synthesis of AgNPs [52,53,54]. The average size of AgCol was estimated using the Debye–Scherrer equation to be about 19.3 nm [46,53]. This observation of the XRD analysis supported the UV–vis and TEM measurements that the bio-constituents in the Col extract played a crucial role in the spherical AgCol stability.

An FTIR analysis was used to confirm the possible interaction between AgCol and the functional groups of capping agents presented in the Col extract. The FTIR spectrum of the Col extract (Figure 5d) reflected intensive peaks of O-H (3261.8 cm^−1^), C-H of alkanes (2925.6 cm^−1^), C=O of esters and ketones (1592.6 cm^−1^), C-O of esters (1394.9 cm^−1^), and C-O of phenolic compounds (1039 cm^−1^) [33]. These characteristic peaks in the Col spectrum were typical of flavonoid compounds, specific anthocyanin molecules. The FTIR spectrum of the AgCol also presented similar absorption peaks at 3437.7, 2925.6, 1629.1, 1383.4, and 1057 cm^−1^, confirming the presence of residual phytochemicals on the surface of the nanoparticles. However, shifts of peaks upon AgCol formation when compared to the Col could be attributed to the reduction of corresponding functional groups. In addition, a marked decrease in the peak intensity was observed at 1057 cm^−1^, indicating a possible involvement of the flavonoid compounds in the reduction process. The FTIR results confirmed that different functional groups of the phytocompounds of the extract were responsible for the formation and stabilization of AgCol, as presented in the previous studies [18,20].

To further confirm the crucial role of bio-compounds in stabilizing and capping the AgCol, TGA was carried out. This authentic technique is often used to determine the thermal stability and decomposition of a sample [19,49]. The TGA thermogram of the AgCol (Figure 5e) exhibited three identical weight loss stages over a wide temperature range from room temperature to 800 ℃. The initial weight loss (1.12%) at 200 ℃ could be involved in the evaporation process of the physically bound water in the AgCol sample. The second weight loss (16.99%) occurred between 200 °C and 600 °C, which could be correlated to decomposition of the bio-organic compounds bound on the surface of the AgCol. The last weight loss at higher temperature could be corresponded to the degradation and evaporation of the metallic silver [55]. Hence, the purity percentage of the AgCol was calculated to be about 81.89 wt% pure silver. The TGA data further confirmed that the presence of organic compounds over the AgCol surface could not only control the rate of reaction but also effectively prevented the agglomeration of nanoparticles, thus, stabilizing them.

**Figure 6:**
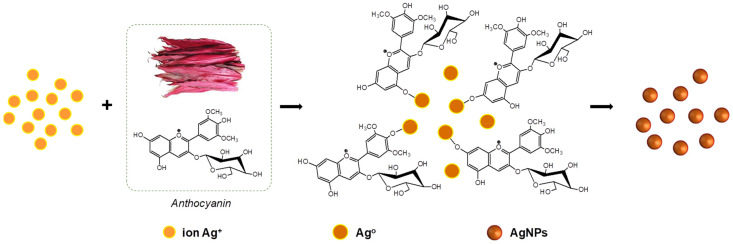
Proposed mechanism for bio-synthesis of AgCol using Col leaf extract.

According to the above analyses, we propose a possible mechanism for bio-synthesis of AgCol using Col leaf extract as shown in Figure 6. Anthocyanin was used as a model molecule because many of bio-molecules existing in the Col leaf extract have similar chemical structures in terms of the presence of hydroxyl groups linked with aromatic rings. The biological compounds that act as a reducing agent may be not necessarily the same which play a role as capping agents. The mechanism of AgCol formation mainly consists of the following three steps: reduction of silver ions to obtain metallic silver atoms, the nucleation of some silver atoms to build up small clusters which act as a template for AgCol formation, and the growth of these clusters and the stabilization of formed AgCol by capping agents to prevent aggregation. This proposed mechanism of AgCol bio-synthesis in the presence of Col leaf extract is in good agreement with previous reports [56,57,58].

### 3.3. Antimicrobial Activity of AgCol

**Figure 7:**
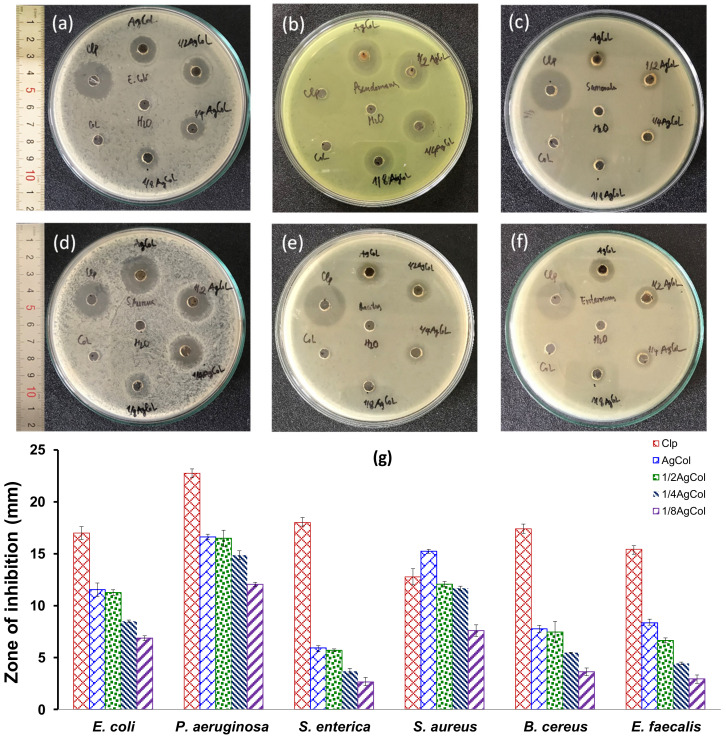
The photographs showing the zone of inhibition of the AgCol against (**a**) *E. coli*, (**b**) *P. aeruginosa*, (**c**) *S. enterica*, (**d**) *S. aureus*, (**e**) *B. cereus*, and (**f**) *E. faecalis*; negative control (H_2_O); positive control (chloramphenicol 200 μg/mL); AgCol (120 μg/mL); 1/2 AgCol (60 μg/mL); 1/4 AgCol (30 μg/mL), 1/8 AgCol (15 μg/mL) and Col extract (10 mg C3G/L); (**g**) quantitative evaluation of antibacterial activity of the AgCol against six pathogenic bacteria (±SD, n = 3).

To assess the antibacterial activity of the AgCol, six different bacterial strains including *E. coli*, *P. aeruginosa*, *S. enterica*, *S. aureus*, *B. cereus*, and *E. faecalis* were exposed to various AgCol concentrations (120, 60, 30, and 15 μg/mL) using the well diffusion method. As shown in Figure 7a–f, the double distilled water (a negative control) and Col extract did not display any antibacterial activity in terms of the ZOI, while the AgCol and the chloramphenicol (a standard antibiotic) demonstrated an obvious ZOI. It was clearly observed that the AgCol possessed promising antimicrobial activities against the tested bacterial pathogens. As presented in Figure 7g, the antibacterial activity was dependent on the tested bacteria strain and the AgCol concentration. Among the tested isolates, the ZOI was found to be the highest against *P. aeruginosa* (16.6 ± 0.27 mm) and the lowest against *S. enterica* (5.9 ± 0.37 mm) at the highest tested AgCol concentration (120 μg/mL). The antimicrobial ability of the AgCol against *E. coli* and *S. aureus* was quite good even at a low concentration (15 μg/mL), with the ZOI of 6.9 ± 0.23 and 7.6 ± 0.56 mm, respectively. Furthermore, the comparison of the ZOI against each bacterial pathogen exhibited the tendency of the enhancement in the antibacterial activity with the increase in AgCol concentration. These bactericidal properties of plant-mediated green synthesized AgNPs have been addressed in previous studies [50,59].

### 3.4. Coloration of the Modified Viscose Fabrics with AgCol and Silk Fibroin

In order to evaluate the adsorption capacity of AgCol on the Vis and VisFib fabrics with change in the wet pickup and AgCol concentration, the colorimetric data (L*, a*, b*), color differences (ΔE*), and color strength (K/S) of the fabric samples were determined, as shown in Table 2. The colorimetric data of both the AgCol-treated Vis and AgCol-treated VisFib fabric samples had a high L* value, and positive a* and b* values, indicating the light browning effect of the AgCol on the fabrics. The ΔE* and K/S values of all AgCol-treated fabric samples increased with the lower wet pickup and the higher AgCol concentration. Furthermore, these values of the AgCol-treated VisFib samples were higher than that of the AgCol-treated Vis samples. This could be explained by the formation of coordination bonding between AgCol and electron-rich nitrogen atoms of amine groups in the fibroin adhered on viscose fabric, which supplemented the linkage of hydroxyl groups of cellulose in viscose fabric with the nanoparticles. Therefore, the fibroin modified viscose fabric could improve the AgCol uptake to compare with the neat viscose fabric. It might enhance antibacterial efficacy and the antibacterial durability of the modified fabrics.

### 3.5. Antibacterial Efficacy of the Modified Viscose Fabrics with AgCol and Silk Fibroin

**Figure 8:**
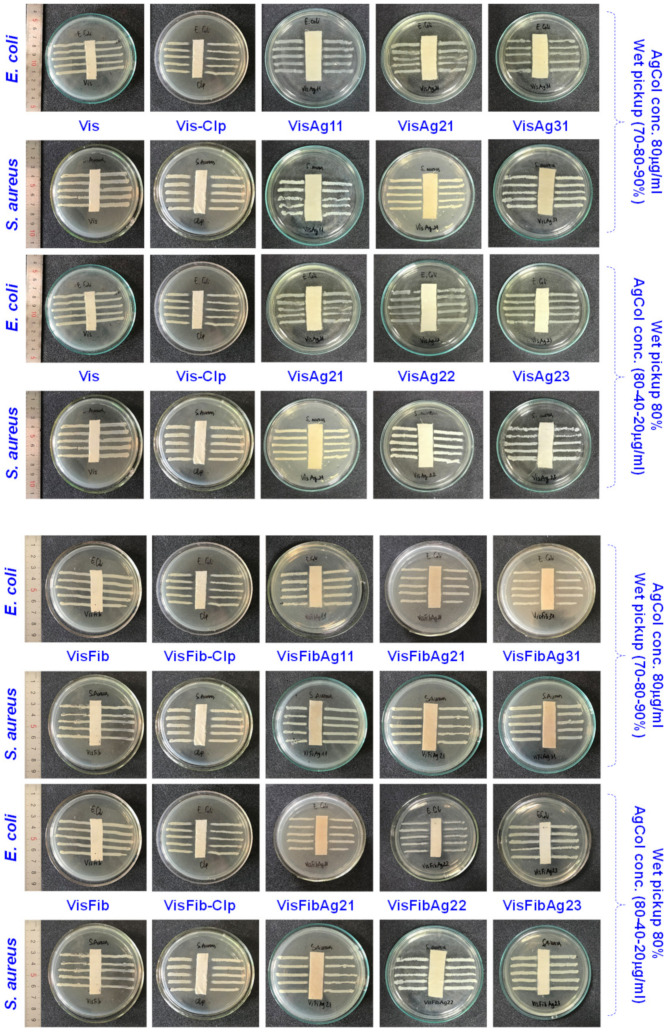
The photographs showing the zone of inhibition of the Vis, VisFib, chloramphenicol-treated (200 μg/mL) Vis (Vis-Clp), and VisFib (VisFib-Clp), and the AgCol-treated fabric samples against *E. coli* and *S. aureus*, with change in the wet pickup and AgCol concentration.

It is well known that AgNPs can endow cellulose fabrics with excellent antibacterial activity [1,3,5]. Thus, antibacterial tests were performed with the AATCC 147-2004 and ASTM E2149-10 methods to investigate the antibacterial efficacy of the AgCol-treated viscose fabrics. As shown in Figure 8, all the AgCol-treated viscose fabrics and the chloramphenicol-treated fabrics revealed an obvious ZOI against *E. coli* and *S. aureus*, while the Vis and VisFib fabrics exhibited no antibacterial activity. This confirms that AgCol-treated viscose fabrics possess antibacterial capability in this work. In addition, the ZOI of AgCol-treated fabric samples seem unchanged with the altering wet pickup, and these were substantially larger with the higher AgCol concentration. Furthermore, these values for the AgCol-treated VisFib samples were higher than those of the AgCol-treated Vis samples. These results demonstrated that the higher the AgCol uptake on fabric samples, the better the antibacterial efficiency. The antibacterial results also gave additional evidence of the enhancement of the AgCol uptake on fabric by the fibroin modifying viscose fabric. The AgCol-treated fabric conditions were chosen to get a good antibacterial efficacy with the wet pickups of 80% and AgCol concentrations of 80 μg/mL. The obtained fabrics under this treatment condition were further evaluated in terms of antibacterial durability against up to 30 home laundering cycles.

**Figure 9:**
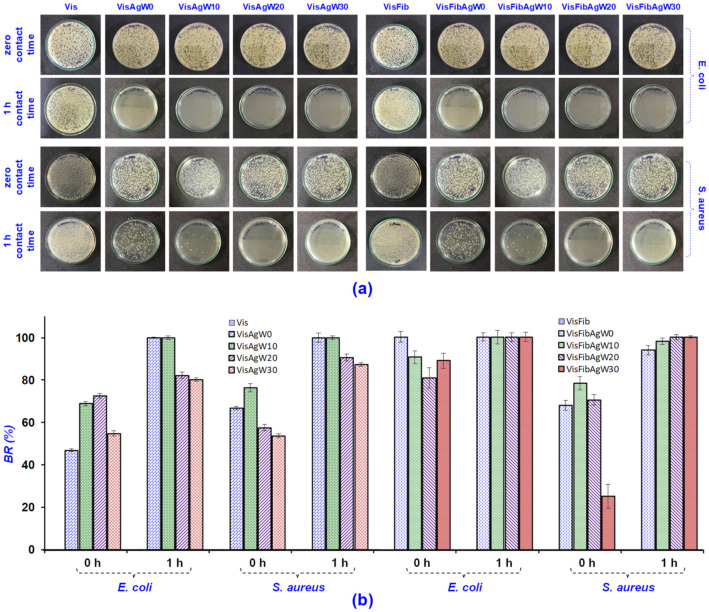
(**a**) The photographs of bacterial growth in the BHI agar plates and (**b**) the antibacterial rate (BR) of the Vis, VisFib, and AgCol-treated fabric samples against *E. coli* and *S. aureus* after 10, 20, and 30 repeated washing cycles.

The durable antimicrobial activity of the AgCol-treated viscose fabrics was qualitatively visualized in Figure 9a, and then plotted in Figure 9b. As shown in Figure 9b, the antibacterial rate (BR) of the VisAg and VisFibAg fabrics for both *E. coli* and *S. aureus* after a 1 h contact period were both 99.99%. This indicated that the AgCol-treated fabrics provide excellent antibacterial properties. The BR values of the VisAg samples for *E. coli* and *S. aureus* were about 80% and 87% after 30 washing cycles, respectively, while these values for the VisFibAg samples still remained 99.99% for both *E. coli* and *S. aureus*.

### 3.6. Characteristics of the Modified Viscose Fabrics with AgCol and Silk Fibroin

**Figure 10:**
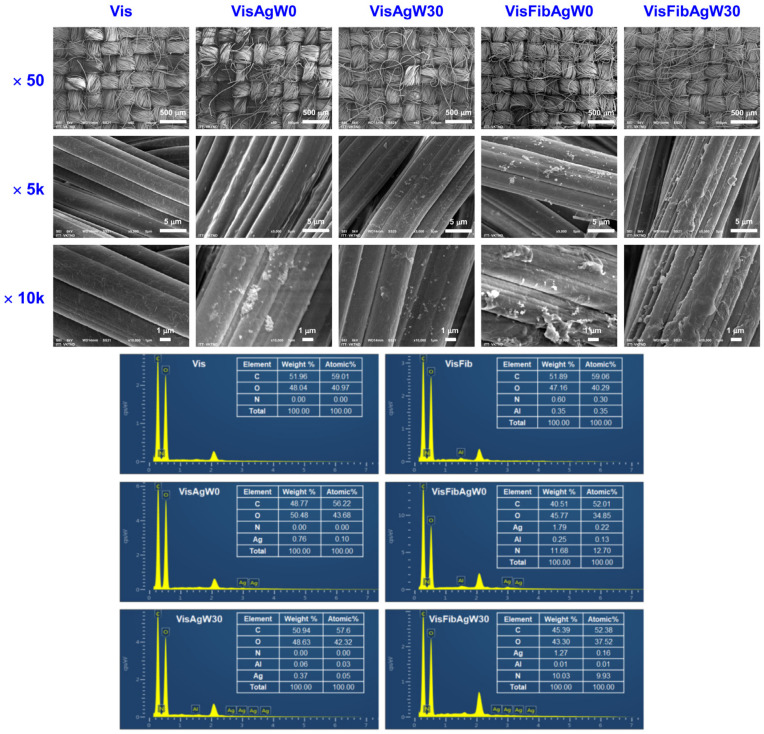
The SEM micrographs at different magnifications of ×50, ×5000, and ×10,000, and EDX spectra of the neat viscose fabric and the AgCol-treated fabrics before and after 30 washing cycles.

To obtain more evidence of the role of fibroin as an effective binder between the AgCol and cellulose fibers in viscose fabric, the fabric surface morphology, AgCol, and fibroin content on the fabrics before and after wash were investigated. The fabric surface morphologies of the original viscose and AgCol-treated viscose fabrics before and after 30 washing tests were explored through SEM at different magnifications, as shown in Figure 10. The untreated fabric displayed the smooth surface of the viscose fibers. Rougher surfaces were observed on the fibers of AgCol-treated fabrics before and after 30 washes, indicating the occurrence of AgCol or/and Fib attached on surface of viscose fibers. The EDX spectra of those fabrics confirmed that the bright points in the SEM images were AgCol. Moreover, the bright points in the SEM micrographs of the VisFibAg fabrics were more than those of the VisAg samples, demonstrating higher AgCol uptake on the VisFibAg fabrics compared with the VisAg fabrics. After 30 washing cycles, the fibroin layer covering the surfaces of viscose fibers was still observed directly in the SEM image of the VisFibAgW30 sample. These results confirmed again that the fibroin acted as binding polymer to coat AgCol on the viscose surface.

The interaction of AgCol and fibroin on the modified fabrics were analyzed by measuring silver and fibroin contents in the treated viscose fabrics before and after 30 washes (Table 3). The silver content of the VisFibAg fabric before washing was 14.3% higher than that of the VisAg fabric. After 30 washing cycles, the loss of silver content was only 25.6% for the VisFibAg fabric, while about 51.2% of silver was released from the VisAg fabric. The fibroin content of the modified fabrics decreased slightly during washing cycles. About 50% of silk fibroin still remained on both the VisFib and VisFibAg fabric after 30 washes, owing to the role of Al^3+^ ions in crosslinking between fibroin molecules and the cellulose fibers of the viscose fabric [41]. The strong linkage between the fibroin and viscose fibers lead to an enhancement in the incorporation of AgCol into the modified fabrics. Due to the presence of numerous amine groups in fibroin, it tends to bind with AgCol by coordination bonds. These results are in harmony and fit well with those illustrated in the SEM micrographs and the antibacterial ability of the AgCol-treated viscose fabrics.

**Figure 11:**
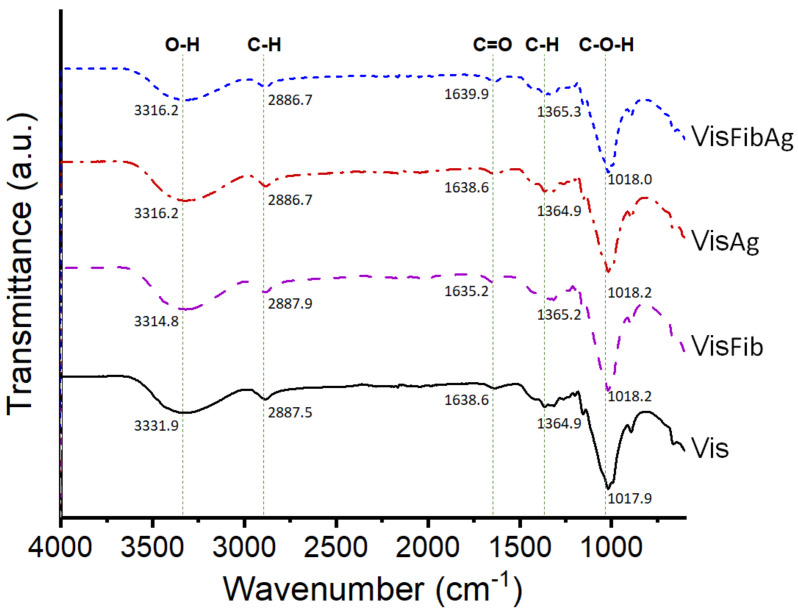
The FTIR spectra of the Vis, VisFib, VisAg, and VisFibAg fabrics.

In order to determine the possible interaction between the functional groups of fibroin on the modified fabric and AgCol, the FTIR measurements of viscose fabric (Vis), fibroin modified viscose fabric (VisFib), AgCol-treated Vis fabric (VisAg), and AgCol-treated VisFib fabric (VisFibAg) samples were carried out, and the spectra were given in Figure 11. The characteristic peaks corresponding to cellulose in the Vis at 3331.9, 2887.5, 1638.6, 1364.9, and 1017.9 cm^−1^ were assigned to the O-H, C-H, C=O, C-H, and C-O-H groups, respectively [41]. Compared to the neat viscose fabric, the VisFib spectrum revealed a fair similarity of these characteristic peaks, with the exception of the lower peak intensities at 3314.8 and 1636.5 cm^−1^ of the VisFib spectrum. It could be explained that the interactions might be occurring via the formation of hydrogen bonding between fibroin’s amide groups and viscose’s hydroxyl groups, and/or via the complexation of Al^3+^ ions with appropriate functional groups of fibroin and viscose [41,60]. The VisAg and VisFibAg spectra also exhibited similar characteristic peaks to those in the Vis and VisFib spectra, respectively, suggesting that the chemical structure of the cellulose was mostly unchanged. However, the peaks of the functional groups in the VisFibAg shifted toward higher wavenumbers at 3316.2, 1639.9, and 1365.3 cm^−1^, and the intensity of these peaks were significantly decreased in comparison with the spectra of the VisFib and VisAg fabrics. The shifting of these peaks occurred due to the interaction of heavy silver atom with amino and amide groups of fibroin molecules, resulting in a decrement in the peak intensity with an ultimate red frequency shift [1,5,31,41].

**Figure 12:**
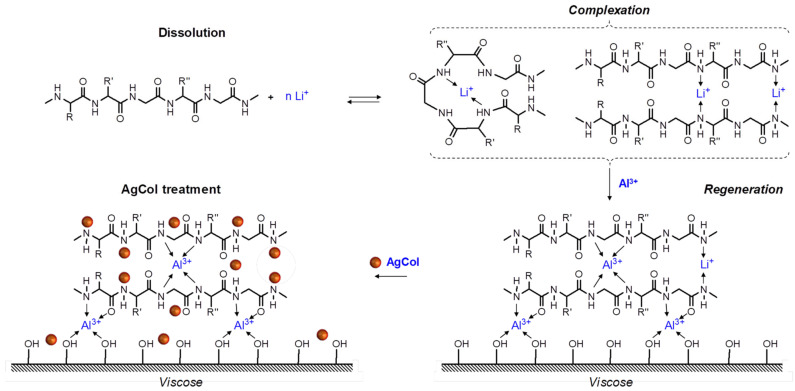
The proposed mechanism of fibroin regeneration and AgCol deposition onto viscose fabric.

On the basis of the literature review of the mechanism of silk fibroin dissolution and regeneration by metal salts [41,60], the complexation ability of fibroin and cellulose with metal ions [61], and the coordination between AgNPs and amine groups [1,5], combining with the experimental results, a proposed mechanism of fibroin regeneration and AgCol deposition onto viscose fabric was elucidated in light of Figure 12. Firstly, the silk fibroin was easily dissolved in the LiEtW solution because lithium halides solutions showed high solvency with silk fibroin [41,60]. The inter- and intra-molecular hydrogen bonds in the chains of fibroin could be broken by the electrophilic attack of the lithium ion that yielded Li-fibroin complexes [41,60]. Secondly, the silk fibroin was regenerated onto the viscose fabric by the ultrafiltration system combining with coagulation of silk fibroin using aluminum salt. Herein, Li^+^ ions in the Li-fibroin complex were replaced by Al^3+^ ions to form a more stable Al-fibroin complex via coordination bonding between Al^3+^ ions and electron-rich nitrogen atoms of amine and amide groups in fibroin. Finally, the fibroin adhered on viscose fabric could enhance the adsorption of AgCol nanoparticles on the modified viscose fabric, and was tightly linked to the fabric via coordination bonding between the silver and the amine and amide groups in fibroin, resulting in the remarkable antibacterial efficacy and laundering durability of the VisFibAg fabric.

## 4. Conclusions

This research provided a novel approach for the fabrication of durable antibacterial viscose fabric modified with bio-synthesized AgCol and silk fibroin as an eco-friendly binder. The AgCol were bio-synthesized using reducing and capping agents from hot water extract of *Cordyline fruticosa* L. leaves. The effect of the reaction conditions, such as the silver salt concentration and the reaction time, were investigated using several analytical techniques including UV–vis, TEM, XRD, FTIR, and TGA. The bio-synthesized AgCol were found to be effective antibacterial activities against six pathogenic bacteria. The AgCol was employed as an antibacterial agent for the functionalization of viscose fabric combined with silk fibroin. The modified viscose fabric with AgCol and fibroin exhibited a remarkable antimicrobial activity against *E. coli* and *S. aureus* bacteria even after 30 washing cycles, due to the presence of numerous amine and amide groups in fibroin, which might form coordination bonding with AgCol. The colorimetry, SEM, EDX, AAS, Kjeldahl, and FTIR analyses provided sufficient evidence for the role of the fibroin as an effective binder between the AgCol and cellulose fibers in viscose fabric. The bio-synthesized AgCol and fibroin modified viscose fabric possessed superior durable antibacterial activity, which would meet the basic criteria for medical textile applications.

## Figures and Tables

**Table 1:** The codes of fabric samples with different treatments.

Sample	Silk Fibroin (%)	AgCol (μg/mL)	Wet Pickup (%)		
			70	80	90
Viscose fabric (Vis)	0	80	VisAg11	VisAg21	VisAg31
	0	40	VisAg12	VisAg22	VisAg32
	0	20	VisAg13	VisAg23	VisAg33
Fibroin treated viscose fabric (VisFib)	2.5	80	VisFibAg11	VisFibAg21	VisFibAg31
	2.5	40	VisFibAg12	VisFibAg22	VisFibAg32
	2.5	20	VisFibAg13	VisFibAg23	VisFibAg33

**Table 2:** Colorimetric data, color differences, K/S value, and images of the AgCol treated and untreated viscose fabric samples, with change in the wet pickup and AgCol concentration.

	Wet Pickup (%)	AgCol (µg/mL)	L*	a*	b*	∆E*	K/S	Fabric Images
Vis	-	-	93.54	−0.96	4.32	0	0.06	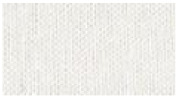
VisFib	-	-	93.47	−0.98	4.93	0.48	0.07	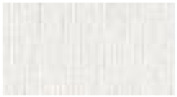
VisAg11	70	80	85.16	3.86	8.95	8.39	0.19	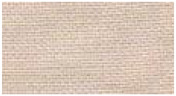
VisAg21	80		84.32	4.55	9.08	9.17	0.2	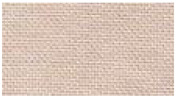
VisAg31	90		85.12	4.1	8.77	8.49	0.18	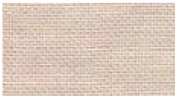
VisAg21	80	80	84.32	4.55	9.08	9.17	0.20	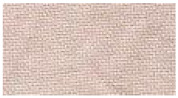
VisAg22		40	85.65	3.15	6.34	6.33	0.15	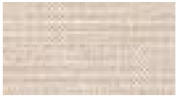
VisAg23		20	89.58	1.11	5.75	3.53	0.11	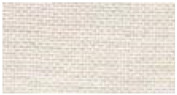
VisFibAg21	80	80	79.95	4.31	6.72	8.91	0.28	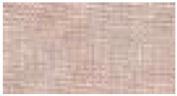
VisFibAg22		40	83.99	2.85	5.90	6.44	0.19	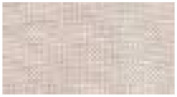
VisFibAg23		20	87.73	1.56	5.22	4.33	0.13	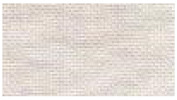

**Table 3:** The silver content, nitrogen content, and fibroin content on the AgCol-treated viscose fabrics before and after 30 washing cycles.

No	Fabric Sample	Washing Cycles	AgCol (μg/mL)	Fibroin (%)	Silver Content (mg/kg)	Nitrogen Content (%)	Fibroin ^a^ Content (%)
1	Vis	-	-	-	-	0.019	-
2	VisFibW0	0	-	2.5	-	0.244	1.406
3	VisFibW30	30	-	-	-	0.121	0.638
4	VisAgW0	0	80	-	642.02	-	-
5	VisAgW30	30	80	-	313.43	-	-
6	VisFibAgW0	0	80	2.5	734.05	0.242	1.394
7	VisFibAgW30	30	80	-	545.86	0.127	0.675

^a^ The protein-to-nitrogen conversion factor is 6.25.

## Data Availability

Not applicable.
